# Stress-Symptom Interaction in Inflammatory Bowel Disease: Longitudinal Digital Phenotyping Study

**DOI:** 10.2196/95854

**Published:** 2026-10-22

**Authors:** Zhihong Li, Yiliminuer Ahemai, Zimei Li, Qianqian Du, Wenwen Zheng, Shaohua Wang, Lazier Halimubai, Yan Wang, Yingbin Cai

**Affiliations:** 1 Second Affiliated Hospital & Yuying Children's Hospital of Wenzhou Medical University, Wenzhou, Zhejiang, China; 2 Xinjiang International Medical Center (Xinjiang International Hospital), Urumqi, China; 3 Department of Gastroenterology, Xishuangbanna Dai Autonomous Prefecture People's Hospital, Yunnan, China; 4 The Third Clinical School of Medicine, Xinjiang Medical University, Urumqi, Xinjiang, China; 5 First Affiliated Hospital of Xinjiang Medical University, Urumqi, Xinjiang, China; 6 Tumor Hospital of Xinjiang Medical University, Xinyi Road Campus, Urumqi, Urumqi, Xinjiang, 830011, China, 86 17799198234, 86 1-416-340-3200

**Keywords:** inflammatory bowel disease, longitudinal digital phenotype, causal emergence, time-varying causal network, module control, central sensitization

## Abstract

**Background:**

Stress and inflammatory bowel disease (IBD) symptoms may vary over short time scales, but these dynamics are not well captured by intermittent clinical assessments.

**Objective:**

The study aimed to characterize exploratory time-varying stress-symptom patterns using intensive longitudinal monitoring and to evaluate a transparent, patient-clustered analysis framework.

**Methods:**

A prospective longitudinal design was used with 74 patients with IBD monitored for 90 days. Daily data comprised 12 core indicators and 2 auxiliary measures. The canonical analysis used patient-level partial-correlation PCMCI (Peter and Clark Momentary Conditional Independence) with a τ of 0 to 2, patient-specific network scaling, a fixed 30-day cycle-end stress contrast, exploratory module-control, and effective-information summaries, and a secondary exploratory time-varying effect model (TVEM).

**Results:**

Mean stress was higher during cycle days 26 to 30 (mean 4.789, SD 1.079) than during nonwindow days (mean 4.160, SD 1.013), with a mean within-person difference of 0.629 (95% CI 0.550‐0.708; *P*<.001); 71 of 74 (95.9%) participants showed a positive difference. Symptom scores had the highest mean of 9.88 (SD 0.09) and lowest dispersion, whereas stressors showed greater variability (mean 3.68, SD 0.49). The estimated network showed predominant forward connections from psychosocial factors through emotion regulation and psychophysiological amplification indicators to gut-specific symptoms. The psychophysiological amplification module had the highest micro-to-macro flow efficiency and macro-level control metric. In the TVEM analysis, the full module-control and causal-emergence models explained 64.4% and 45.6% of the deviance, respectively.

**Conclusions:**

In this longitudinal IBD patient cohort, daily monitoring identified a reproducible end-of-cycle stress comparison and an exploratory multiscale organization that links psychosocial, emotional, psychophysiological, and gut-specific symptom domains. The psychophysiological amplification module provides the most informative, comprehensive representation, although its organization varies depending on follow-up and disease status. These findings support the use of digital phenotypes to generate clinical evidence for the coordination of stress symptoms, tailored to individual situations.

## Introduction

Inflammatory bowel disease (IBD) is a chronic gastrointestinal (GI) disease characterized by a relapsing-remitting course, comprising Crohn disease and ulcerative colitis, and it imposes a substantial global health and socioeconomic burden [1,2]. In addition to the hallmark GI manifestations (eg, abdominal pain, diarrhea, and rectal bleeding), IBD is increasingly recognized as a systemic disease [3] characterized by heterogeneous GI and extraintestinal manifestations, with psychological stress increasingly recognized as a key moderator of symptom severity and risk of recurrence [4,5], which often exacerbates symptoms and induces disease recurrence. The complexity of these phenotypes is further compounded by the highly dynamic nature of the disease, and clinical management of IBD is currently hampered by 2 interrelated challenges: a lack of mechanistic understanding of disease dynamics and an inability to identify actionable, patient-specific targets for intervention [6,7]; despite advances in biologic agents and small-molecule therapies [8-10], the unpredictable switch between remission and relapse, combined with stress-related fluctuations in symptoms, remains a major clinical challenge. Network-based simulations and personalized time-series models have also been proposed as approaches for generating patient-specific hypotheses and identifying actionable intervention targets [11,12]. This limitation is exacerbated by reliance on cross-sectional or sparsely sampled longitudinal data, which masks the temporal dynamics of symptom evolution and causal relationships between disease-related variables [12,13].

Recent advances in digital health technologies, including wearable sensors, mobile health (mHealth) apps, and continuous physiological monitoring, have allowed us to collect dense longitudinal microdata (eg, daily symptom scores, physiological parameters, and behavioral metrics) with unprecedented temporal resolution [12,13], which offer a unique opportunity to characterize IBD and reveal the complex causal networks underlying disease activity. Translating this wealth of data into actionable clinical insights will require integrated analytic frameworks that can effectively decipher the complex interconnections among stress, symptoms, and disease dynamics to support precision decision-making. Current clinical practice and research increasingly need tools that can connect data collection and clinical applications to achieve a holistic understanding of disease progression. Comprehensive approaches that integrate causal inference [14,15], network science [16], and time-varying modeling [17] are essential to identify key regulatory modules, verify their clinical relevance, and track dynamic changes. This framework can not only unlock the potential of longitudinal microdata but also fit the core goal of precision medicine in IBD to provide targeted interventions for each patient at the right time.

This study aimed to develop and evaluate an integrative longitudinal analysis framework for characterizing the interaction between stress and IBD symptoms. The objectives were to describe time-lagged and instantaneous relationships among stress, cognitive behavioral, psychophysiological, and symptom variables; to identify functional network modules; and to evaluate dynamic changes in causal-emergence and module-control metrics.

## Methods

### Study Design and Participants

This study used a prospective longitudinal design to collect high-density time-series data from patients with IBD, with the aim of evaluating the proposed integrative analysis framework. The overall analytic workflow is summarized in Figure 1. Eligible participants had a diagnosis of IBD, were 18 to 65 years of age, had a disease duration of 6 months or more, and had disease activity within the prespecified baseline eligibility ranges (Mayo score of 2-4 for ulcerative colitis and Simplified Endoscopic Activity Score for Crohn Disease [SES-CD] score of 3-7 for Crohn disease). The Mayo score and SES-CD are disease-specific instruments; their raw scores were not treated as directly interchangeable measures across IBD subtypes. Exclusion criteria included serious mental disorders requiring systematic treatment (eg, schizophrenia, bipolar disorder); use of glucocorticoids, immunosuppressants, or biological agents within 1 month before enrollment; other serious GI diseases (such as gastric cancer and intestinal obstruction); or inability to complete daily data recording or use study equipment.

A total of 74 patients with IBD were recruited from the First Affiliated Hospital of Xinjiang Medical University from January 2024 to March 2025. The follow-up period was 90 days (days 0‐89). Clinical visits were scheduled at baseline (day 0), day 45, and day 89 for standardized assessments and data verification. Disease activity was characterized using the disease-specific instrument for each subtype: the Mayo score for ulcerative colitis and the SES-CD for Crohn disease. These measures were used as subtype-specific clinical covariates and were not combined into a common raw disease activity score.

**Figure 1.**
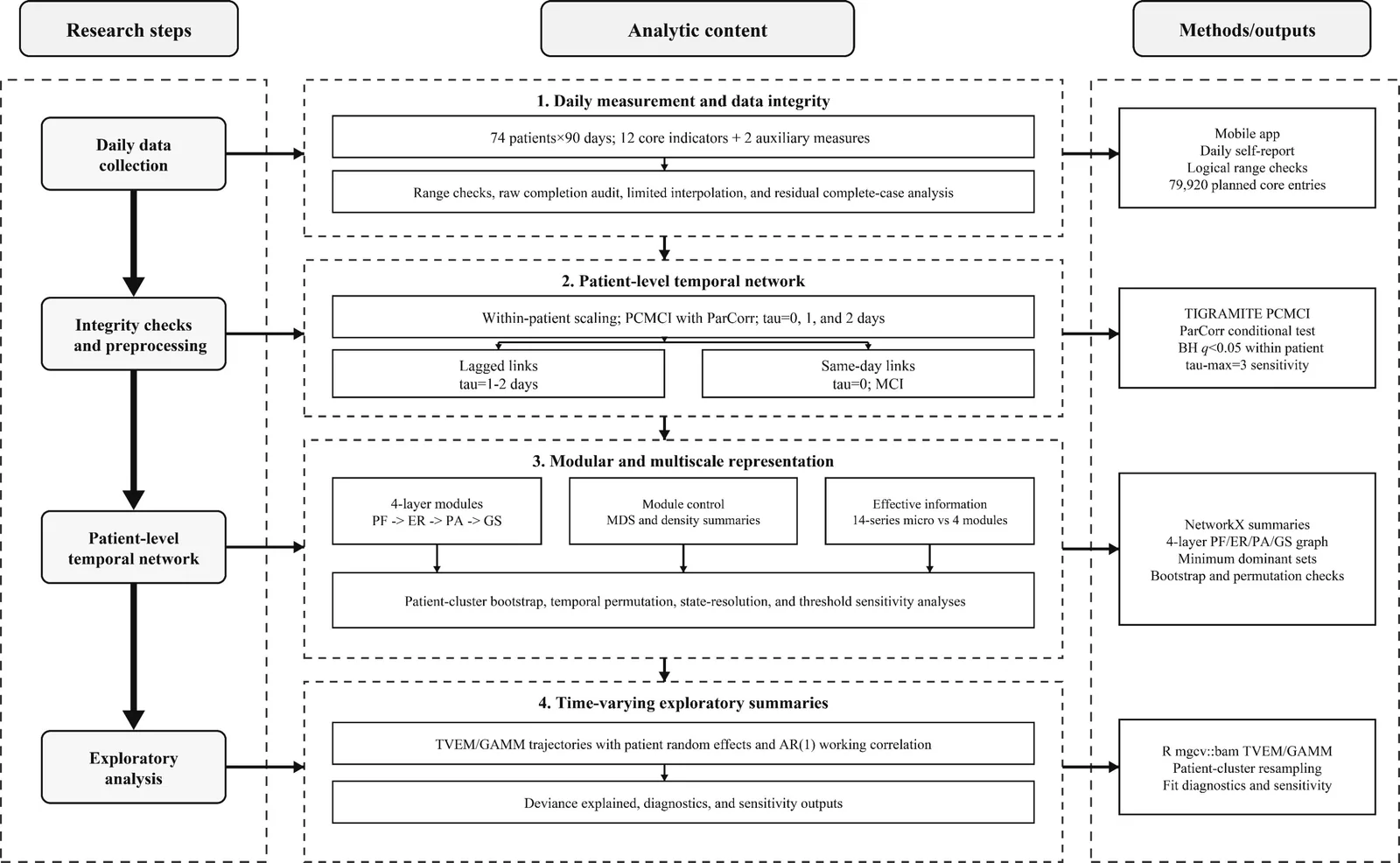
Methods workflow. The diagram summarizes the analytic sequence from daily data collection and integrity checks to patient-level temporal network estimation, modular and multiscale representation, and exploratory time-varying summaries. AR(1): first-order autoregressive; BH: Benjamini-Hochberg; ER: emotion regulation; GAMM: generalized additive mixed models; GS: gut-specific symptoms; MCI: momentary conditional independence; ParCorr: partial correlation; PA: psychophysiological amplification; PCMCI: Peter and Clark Momentary Conditional Independence; PF: psychosocial factors; TVEM: time-varying effect model.

### Data Collection and Variable Definitions

High-density time-series data were collected daily for 90 consecutive days. To make the analysis set explicit, the daily measures were organized into 12 core indicators across 4 domains: psychosocial factors (PF: work stress, health worry, and social conflict), emotion regulation (ER: rumination, catastrophizing, and avoidance), psychophysiological amplification (PA: cognitive anxiety, somatic anxiety, and negative affect), and gut-specific symptoms (GS: abdominal pain, bloating, and bowel urgency). Information seeking and fatigue were collected as 2 auxiliary daily measures and mapped to ER and PA, respectively. These auxiliary measures were not counted in the 12-indicator denominator or the 94.3% (75,365/79,920) core completion calculation, but they were retained in explicitly exploratory network and module analyses. Accordingly, Figure 2 displays 14 nodes or dimensions (12 core indicators+2 auxiliary measures). The source instruments, score handling, and analysis roles of all core and auxiliary measures are specified in Table S2 in Multimedia Appendix 1. For stressor assessment, the brief Perceived Stress Scale, 10 item (PSS-10) [18,19] was used to assess daily stress levels, and the 90-day average daily score was calculated. The cycle-end window was prespecified as a window for exploratory dynamic analysis and was defined as cycle days 26 to 30 of each 30-day study cycle. We calculated each participant’s mean stress score within and outside this window and estimated the participant-level difference with a 2-sided 1-sample *t* test and 95% CI. We also fitted a linear mixed effects model with daily stress score as the outcome, cycle-end window and study day as fixed effects, and a participant-specific random intercept. This analysis quantified a prespecified window contrast; it was not a test of full periodicity and did not establish that the window represented each participant’s maximum stress. The 79,920 figure refers to the planned core variable-day matrix (74 participants×90 days×12 core indicators), whereas the 94.3% (75,365/79,920) completion rate refers to raw core entries before preprocessing. For each variable, interpolation was permitted only when raw missingness was at most 5% and only across internal gaps of at most 2 observations; variables with completion below 92% were flagged, and residual gaps were analyzed on a complete-case basis. Cognitive behavioral factors were assessed using the Cognitive Emotion Regulation Questionnaire (CERQ) [20] and the Health Information Seeking Behavior Scale (HISBS) [21]. The Hospital Anxiety and Depression Scale (HADS) [22], Positive and Negative Affect Schedule (PANAS) [23], and 20-item Multidimensional Fatigue Inventory (MFI-20) [24,25] were used to evaluate psychological and fatigue-related measures. IBD symptoms were evaluated using the study-labeled Inflammatory Bowel Disease Symptom Score Scale (IBD-SI) [26], including bowel urgency, bloating, and abdominal pain. Raw daily data were retained for the primary analyses; any 7-day smoothing used in descriptive figures was labeled as visualization-only and was not used in inferential models. Medical events reported during follow-up were recorded when available, but treatment changes, infections, and flare status were not systematically captured as daily time-varying covariates. Objective inflammatory measures, including endoscopic activity, C-reactive protein, and fecal calprotectin, were not systematically available across the follow-up and were not included as fully time-varying covariates. Sleep, diet, menstrual-cycle effects, and medication adherence were also not systematically collected for model adjustment. Daily data were collected with a customized mHealth application with built-in logical checks to ensure data validity (eg, scoring-range limits). Missing data were handled by a prespecified variable-level policy: interpolation was permitted only when raw missingness was at most 5% and only for internal gaps of at most 2 observations; variables with completion below 92% were flagged; and residual gaps were analyzed on a complete-case basis. Baseline data were collected using a structured questionnaire, medical record review, and clinical assessment.

**Figure 2.**
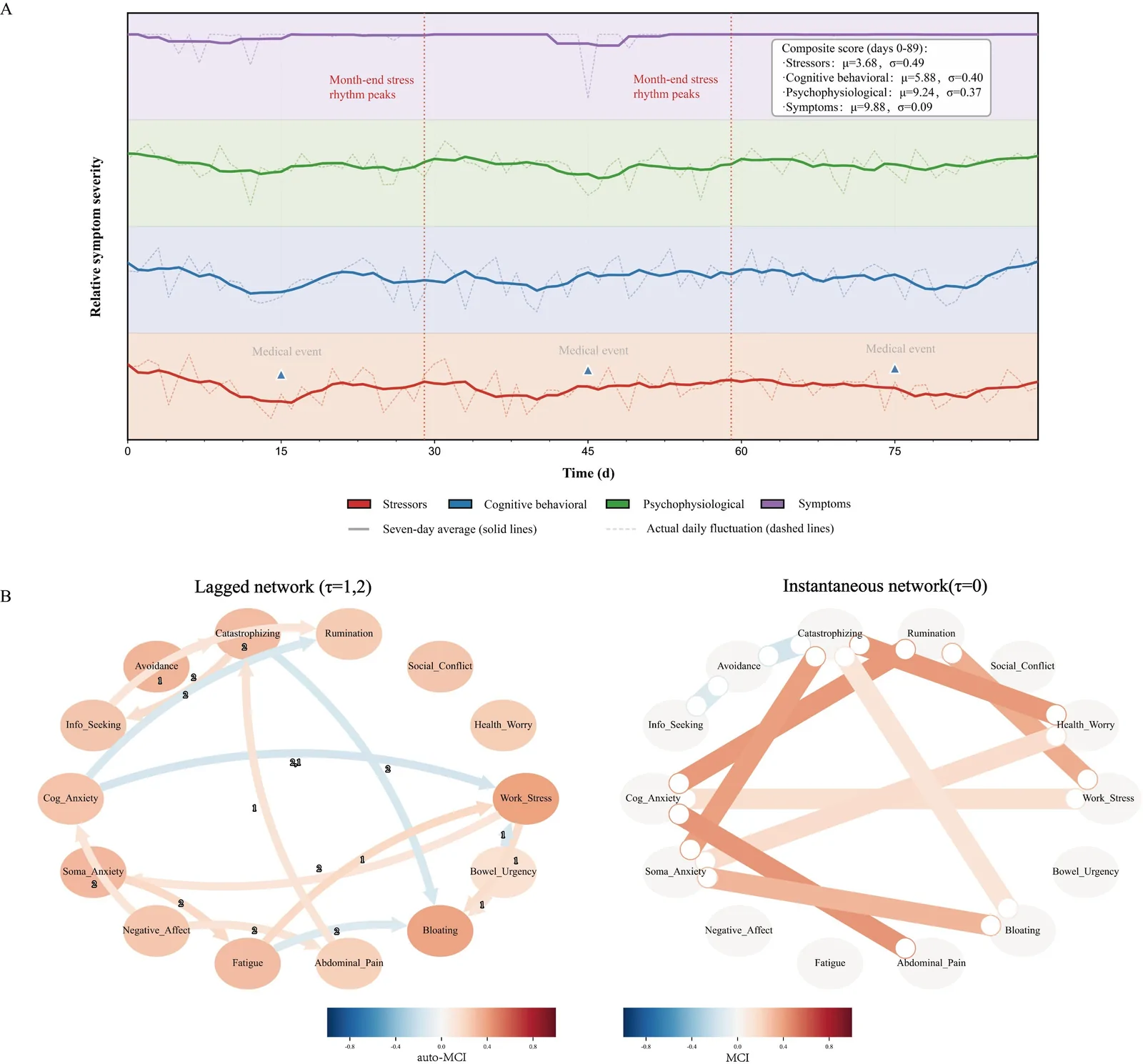
High-resolution longitudinal monitoring describes temporal coupling between stress patterns and symptom severity in a representative patient with inflammatory bowel disease. (A) Daily symptom severity trajectories (relative score) over 90 days, showing temporal coincidence in the displayed individual series with a prespecified cycle-end window (cycle days 26‐30). Blue and green shaded areas demarcate periods of symptom exacerbation and attenuation, respectively, in the displayed time series. The table inset summarizes composite scores (mean, SD) across 4 key dimensions. The legend distinguishes the 7-day average random-volatility value from the actual daily data fluctuation; both labels were retained and strengthened for readability. (B) Model-derived conditional-association networks among the displayed variables. The lagged network (left) depicts estimated 1- to 2-day temporal associations, while the instantaneous network (right) captures same-day associations. Functional domains are identified by node labels and panel layout; node fill is not used as a domain key. Arrows indicate the direction and relative strength of estimated links. The lagged network uses the auto-MCI color scale, whereas the instantaneous network uses the MCI color scale. Cog_Anxiety: cognitive anxiety; Info_Seeking: information seeking; MCI: momentary conditional independence; Som_Anxiety: somatic anxiety.

### PCMCI and Group-Level Temporal Network Summary

Time-lagged and contemporaneous conditional associations among the daily series were estimated using the exploratory PCMCI (Peter and Clark Momentary Conditional Independence) algorithm [27] and the Tigramite package (Causal Inference Lab). PCMCI is a 2-stage time-series procedure: the principal component 1 phase removes conditionally independent candidate links, and the momentary conditional independence phase tests the remaining lagged links while conditioning on relevant past variables. PCMCI was fitted separately for each eligible patient using the analytic partial-correlation (ParCorr) conditional independence test, with τ of 0, 1, and 2 days and Benjamini-Hochberg *q*-values controlled at 0.05 within each patient. The primary lag window was limited to 0 to 2 days to represent short-term carryover that can be resolved by daily assessments while also limiting parameter growth and the sparsity of effective transitions in the 90-day patient-level series. This restriction does not imply that longer delays are absent; a τ-max sensitivity analysis through 3 days is reported in Table S5 in Multimedia Appendix 1. The patient, rather than the person-day, was the independent unit. Network inputs were standardized within patients after the prespecified missing-data step. These outputs are directed, model-derived conditional associations under linear and stationarity assumptions; they do not establish experimental causality, biological mechanisms, mediation, or intervention responsiveness. Patient-cluster bootstrap intervals summarize support and signed direction across fitted patient-level links; the bootstrap resamples patients after PCMCI fitting and does not refit PCMCI. Lag-order and alpha-threshold sensitivity grids are reported in Multimedia Appendix 1. NetworkX was used for exploratory centrality summaries.

We integrated model-derived phase, entropy, and energy metrics to describe exploratory changes in directed temporal network structure. Based on disease trajectory patterns, participants were categorized into 3 subgroups. We compared network metrics across subgroups, identified candidate transition boundaries and symptom-feedback nodes, and tracked changes in model-derived phase trajectories during disease-state transitions. Positive Causal Entropy was interpreted as the uncertainty in forward temporal transmission, whereas Reverse Causal Energy was interpreted as symptom-related feedback intensity acting on upstream variables.

### Network Structure and Module Control Analysis

Based on the directed temporal network estimated using exploratory PCMCI, a 4-layer modular network representation was constructed. The input layer (PF) contained the 3 core PF indicators; the processing layer (ER) contained the 3 core ER indicators plus auxiliary information seeking; the amplification layer (PA) contained the 3 core PA indicators plus auxiliary fatigue; and the output layer (GS) contained the 3 core GS indicators. The resulting module-control metrics are exploratory summaries of a thresholded, model-derived graph. Minimum dominant sets were defined as the smallest sets of driver nodes that collectively reached all module nodes in the thresholded graph. Driver nodes, controllability proxies, module flows, and threshold sensitivity were calculated with the patient as the resampling unit.

### Effective-Information and Causal-Emergence Metrics

Exploratory multiscale information analysis compared a 14-series micro representation with 4 prespecified macro modules. Each micro variable was converted to 2 within-patient rank categories; each macro-state was the rounded mean category of its assigned variables. Observed adjacent transitions were counted without crossing patient boundaries. Transition probabilities were estimated with additive smoothing of 0.50 and a uniform intervention distribution across the represented current states. Effective information was reported in bits, and causal-emergence strength was defined as macro effective information minus micro effective information. A patient-cluster bootstrap, within-patient temporal permutation, and sensitivity to 2-category vs 3-category state resolution were prespecified.

### Time-Varying Effect Model

A secondary exploratory time-varying effect analysis was specified only for daily model-derived outcomes supplied in the analysis input. In R, mgcv::bam used cubic regression splines with primary fixed day knots at 0, 10, 25, 50, 65, 75, and 89 (including cycle landmarks 25, 50, and 75), a patient-level random intercept, and a patient-level first-order autoregressive (AR(1)) working correlation. The AR(1) rho estimate, based on ordered within-patient outcome residual proxies, was recorded in the output manifest. Models were adjusted for IBD subtype, age, sex, disease duration, and within-patient *z*-scored disease activity and HADS. Sensitivity models varied the time-basis dimension and used a scaled-t outcome family. All time-varying effect model (TVEM) results were exploratory descriptions of model-derived outcomes and were not interpreted as treatment effects, biological mechanisms, or causal trends.

Because the independent sample consisted of 74 patients and the downstream models were exploratory, we did not treat the large collection of module, subtype, and time-varying terms as confirmatory hypothesis tests. The sensitivity output reports basis-dimension comparisons, scaled-t alternatives, effective degrees of freedom, residual diagnostics, and dependence parameters.

### Covariate Adjustment

To assess potential confounding of the relationships between the core variables and the model-derived outcomes, study participants were stratified according to key categorical covariates, and differences in network structure and module metrics between subgroups were compared at each level. Generalized additive mixed models were used to quantify the contribution of the available covariates, with adjustment for individual random effects and time-series autocorrelation. This covariate strategy does not eliminate residual confounding by clinical, treatment-related, behavioral, or physiological factors that were not systematically measured.

### Statistical Analysis

The reproducibility repository contains a pinned Python (version 3.11; Python Software Foundation) environment and an R (version 4.3.1; R Foundation for Statistical Computing) environment description. The canonical Python analysis uses analytic Tigramite ParCorr with a τ of 0 to 2, patient-specific within-person *z*-scores, patient-level Benjamini-Hochberg control, and patient-cluster stability summaries. The primary cycle-end contrast uses raw stress scores and fixed 30-day study cycles. Network, module-control, causal-emergence, and TVEM analyses are exploratory; all resampling uses patients as the independent unit.

### Ethical Considerations

This study was reviewed and approved by the Ethics Committee of the First Affiliated Hospital of Xinjiang Medical University (approval number K202412-86). All participants provided written informed consent before enrollment. No directly identifiable participant information is presented in this study.

## Results

### Study Cohort

The planned core microdynamic matrix comprised 79,920 variable-days (12 core indicators×90 days×74 participants). Before interpolation and exclusion, the raw core completion rate was 94.3% (n=75,365). Information seeking and fatigue were auxiliary measures and were excluded from both the denominator and the completion calculation; the exploratory network, module-control, and multiscale information analyses nevertheless retained these 2 measures, yielding 14 input series. The baseline demographic and clinical characteristics of the 74 participants are summarized in Table 1.

**Table 1.** Baseline demographic and clinical characteristics of participants with inflammatory bowel disease^a^.

Variables	Overall (N=74)	CD^b^ (n=40)	UC^c^ (n=34)	*P* value
Sex, n (%)				.26
Male	39 (53)	24 (60)	15 (44)	
Female	35 (47)	16 (40)	19 (56)	
Degree of education, n (%)				.14
Junior high school and below	16 (22)	8 (20)	8 (24)	
High school/technical secondary school	32 (43)	14 (35)	18 (53)	
College or above	26 (35)	18 (45)	8 (24)	
Marital status, n (%)				>.99
Married	59 (80)	32 (80)	27 (79)	
Unmarried/divorced/widowed	15 (20)	8 (20)	7 (21)	
Status of occupation, n (%)				.27
Incumbency	53 (72)	26 (65)	27 (79)	
Separation or retirement	21 (28)	14 (35)	7 (21)	
Have children, n (%)				.33
Yes	65 (88)	37 (92)	28 (82)	
No	9 (12)	3 (8)	6 (18)	
Family history, n (%)				.85
Yes	5 (7)	2 (5)	3 (9)	
No	69 (93)	38 (95)	31 (91)	
Extraintestinal manifestations, n (%)				.96
Multiple complications	8 (11)	4 (10)	4 (12)	
Single complication	16 (22)	9 (22)	7 (21)	
No	50 (68)	27 (68)	23 (68)	
Presence of stoma, n (%)				>.99
Yes	7 (10)	4 (10)	3 (9)	
No	67 (90)	36 (90)	31 (91)	
30-day stressful events, n (%)				.16
Yes	20 (27)	14 (35)	6 (18)	
No	54 (73)	26 (65)	28 (82)	
Age (y), mean (SD)	43.68 (8.12)	42.36 (7.18)	45.32 (8.97)	.13
Disease course (y), mean (SD)	7.65 (2.99)	8.20 (2.97)	7.00 (2.97)	.09
Disease activity: SES-CD^d^ (CD only), mean (SD)	5.48 (1.52)	5.48 (1.52)	—^e^	Descriptive only
Disease activity: Mayo score (UC only), mean (SD)	4.78 (1.05)	—^e^	4.78 (1.05)	Descriptive only
HADS^f^ score, mean (SD)	8.18 (2.32)	8.92 (2.32)	7.32 (2.02)	.002
Pressure perception, mean (SD)	5.43 (1.25)	5.58 (1.36)	5.25 (1.10)	.25
Cognitive behavior (daily composite score), mean (SD)	5.98 (0.89)	5.90 (0.96)	6.07 (0.80)	.42
Psychophysiology (daily composite score), mean (SD)	9.42 (1.38)	9.84 (1.47)	8.92 (1.10)	.003
Symptom (daily composite score), mean (SD)	10.04 (1.13)	10.19 (1.09)	9.87 (1.18)	.23

a For the disease-specific rows, the overall column reports the mean (SD) values within the relevant subtype (CD for SES-CD and UC for Mayo score), and the corresponding subtype column reports the same value. The dash indicates that the instrument was not applicable to the opposite subtype rather than that data were missing.

b CD: Crohn disease.

c UC: ulcerative colitis.

d SES-CD: Simplified Endoscopic Activity Score for Crohn Disease.

e Indicates that the instrument was not applicable to the opposite subtype rather than that data were missing.

f HADS: Hospital Anxiety and Depression Scale.

### Individual Stress-Symptom Dynamics

Through in-depth analysis of 90 days of high-density monitoring data from 74 participants, we quantified stress differences between the prespecified cycle-end window and nonwindow days. A representative 90-day symptom trajectory and the corresponding model-derived lagged and instantaneous networks are shown in Figure 3. The analytic dataset contained 6660 participant-days, including 1110 cycle-end-window observations and 5550 nonwindow observations. Mean stress was 4.789 (SD 1.079) during cycle-end days 26 to 30 and 4.160 (SD 1.013) outside the window. The mean participant-specific difference was 0.629 (95% CI 0.550‐0.708 points; *P*<.001), and 71 of 74 (95.9%) participants had a positive cycle-end minus noncycle-end difference. In a linear mixed effects model adjusted for study day and including a participant-level random intercept, the cycle-end-window coefficient was 0.608 (95% CI 0.553‐0.663; *P*<.001). These results support higher average stress during the prespecified window but do not establish an individual-specific maximum or a recurring population-wide monthly rhythm. The corresponding individual-level distributions and daily trajectory are provided in Table S1 and Figure S1 in Multimedia Appendix 1. The remaining network findings are interpreted as model-derived temporal associations and do not establish mediation or biological causality. The symptom dimension exhibited the highest mean (μ=9.88) and extremely low dispersion (σ=0.09), indicating high homogeneity in symptom burden across the cohort. In contrast, stressors (μ=3.68, σ=0.49) and psychophysiological indicators (μ=9.24, σ=0.37) exhibited greater interindividual variability.

**Figure 3.**
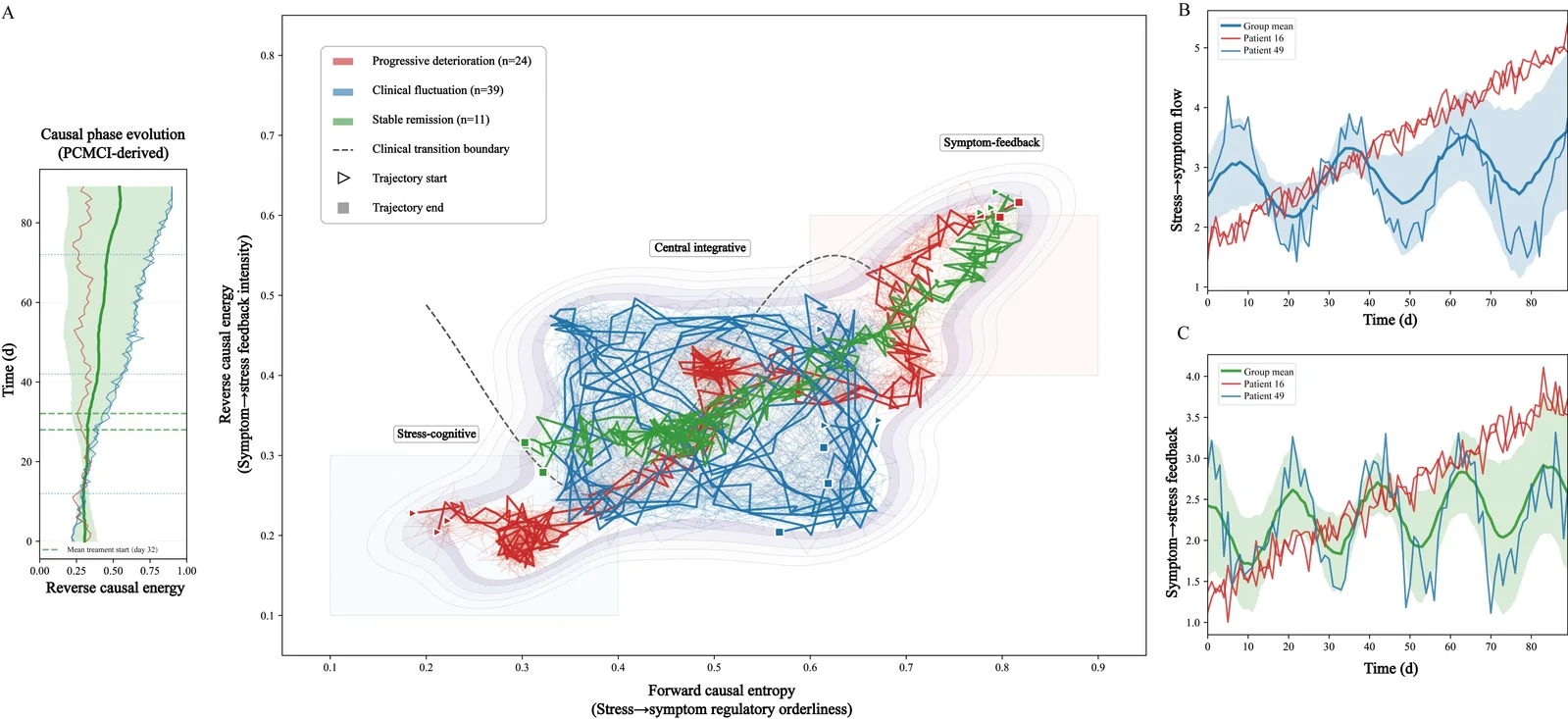
Exploratory patient-level network summaries and sensitivity results. (A) Peter and Clark Momentary Conditional Independence (PCMCI)–derived patient trajectories in forward causal entropy vs reverse causal energy space, with trajectory classes, the transition boundary, and the time course of reverse causal energy shown in the inset. (B) Adjusted stress-to-symptom module flow across the 90-day observation period for the group mean and 2 illustrative patients. (C) Adjusted symptom-to-stress feedback across the follow-up period for the group mean and 2 illustrative patients. Lines and shaded bands represent model-derived estimates and pointwise uncertainty summaries; the treatment-initiation marker is descriptive.

### Group-Level Temporal Network Summary

The exploratory network analysis was summarized at the patient level rather than being interpreted as a stable population mechanism. We report the number of eligible patients, patient support, signed direction agreement, and cluster bootstrap intervals for retained links and module summaries. Any time-varying pattern shown in Figure 4 is a model-dependent descriptive summary and may reflect inflammation, treatment, reporting behavior, or other unmeasured influences.

**Figure 4.**
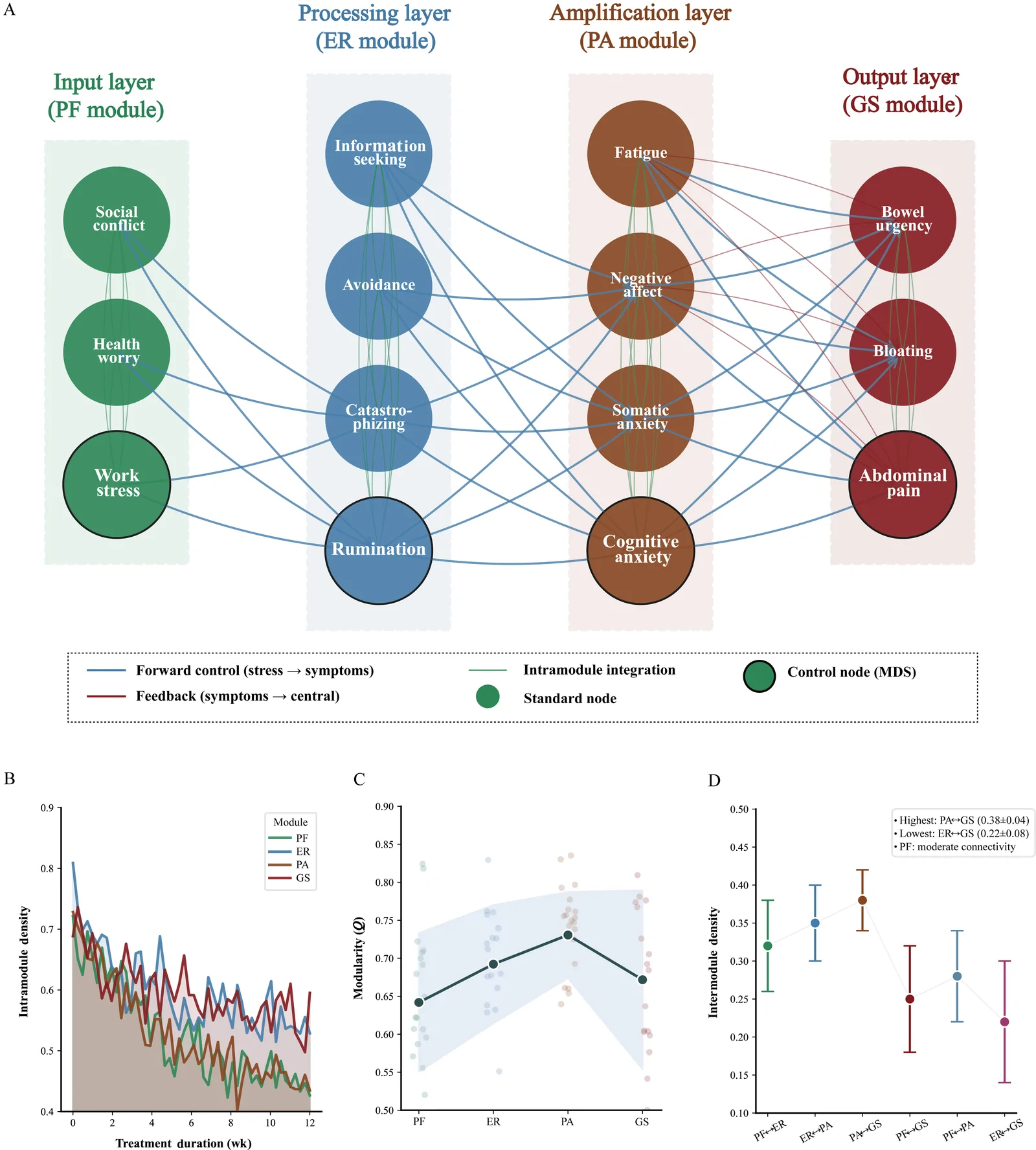
A hierarchical modular network representation summarizes the exploratory organization of inflammatory bowel disease symptom–related variables. (A) The 14 displayed nodes comprise 12 core indicators and 2 auxiliary measures: information seeking in emotion regulation (ER) and fatigue in psychophysiological amplification (PA). The 4-layer network extends from psychosocial factors through ER and PA to gut-specific symptoms (GS); edge colors distinguish forward control, feedback, and intramodule integration, and outlined nodes denote model-derived control nodes. (B) Intramodule connection density across treatment duration. (C) Module-level modularity (*Q*) with patient-level distributions and the group summary. (D) Intermodule connection density for psychosocial factors (PF)-to-ER, ER-to-PA, PA-to-GS, PF-to-GS, PF-to-PA, and ER-to-GS interfaces. MDS: minimum dominant set.

### Module Control Network Analysis

Network analysis identified 4 functionally defined modules organized sequentially for information processing (Figure 4A). The exploratory representation contained 14 nodes: 12 core indicators and 2 auxiliary measures. The input layer (PF module) contained work stress, health worry, and social conflict; the processing layer (ER module) contained rumination, catastrophizing, and avoidance plus auxiliary information seeking; the amplification layer (PA module) contained cognitive anxiety, somatic anxiety, and negative affect plus auxiliary fatigue; and the output layer (GS module) contained abdominal pain, bloating, and bowel urgency. Connections between modules predominantly showed a feedforward temporal pattern from stress-related cognition toward emotional and symptom variables. Feedback connections from the output layer to the amplification layer were also identified, consistent with a possible reciprocal association between somatic symptoms and emotional or psychophysiological states. Within modules, high-density integrative connections and core-dominant nodes represented model-derived information convergence. Intramodule connection density showed an overall upward trend during follow-up (Figure 4B). The ER and PA modules had the highest modularity *Q* values (Figure 4C), indicating greater relative separation within this exploratory network. Intermodule connection density was highest from PF to ER, followed by PA to GS (Figure 4D).

### Causal-Emergence Summary

The exploratory module analysis summarized the psychophysiological amplification module as a model-derived integrative unit in the thresholded model-derived graph. The corresponding module-level control and effective-information metrics are shown in Figure 5. The label describes the functional grouping of measured affective, cognitive, and fatigue-related variables; it is not a validated central-sensitization or pain-amplification construct. Module metrics, effective-information contrasts, patient-cluster bootstrap intervals, temporal permutation results, and state-resolution sensitivity are reported without assigning biological or intervention meaning.

**Figure 5.**
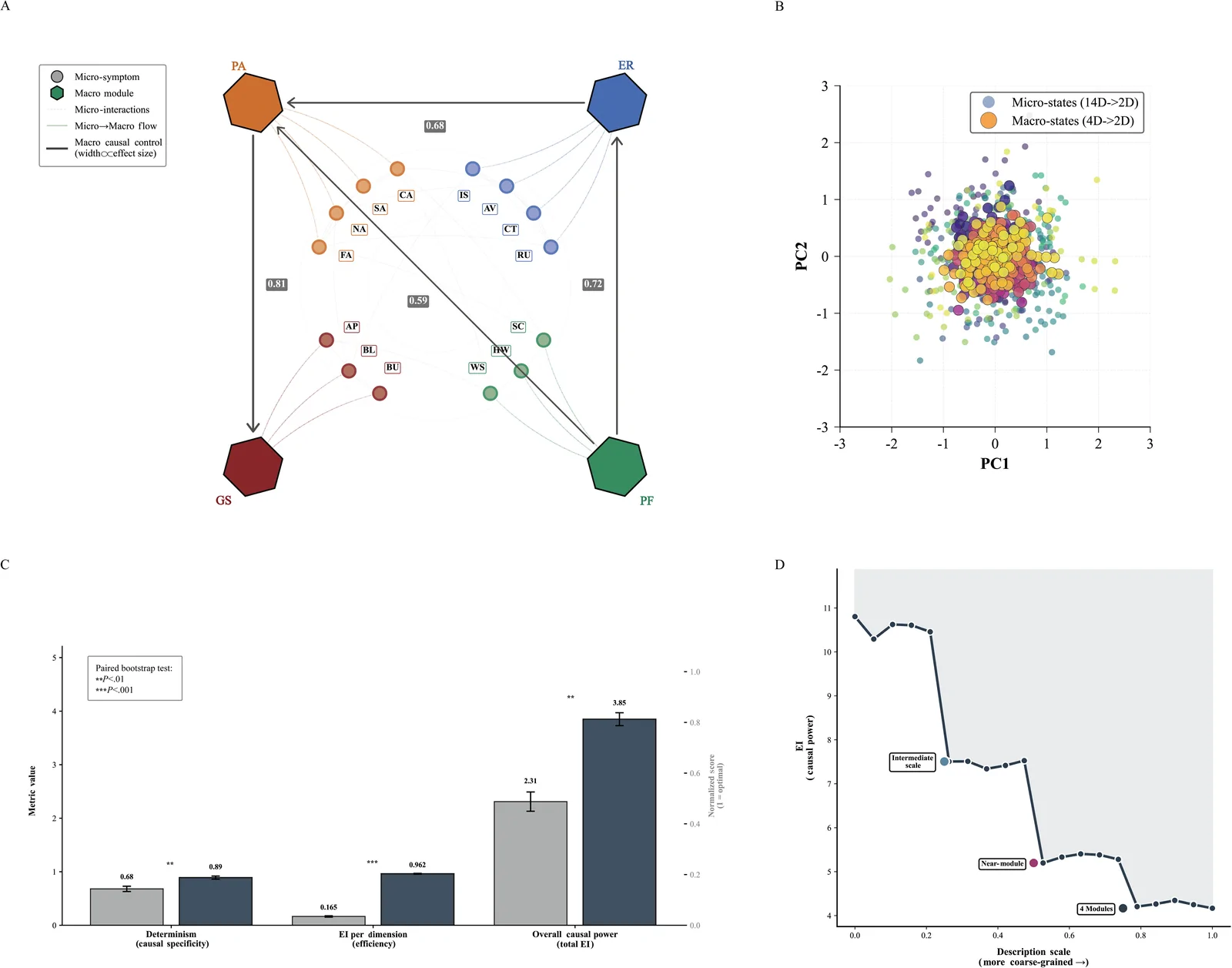
A model-derived multiscale information metrics identify the psychophysiological amplification (PA) module as a model-derived integrative unit. (A) Module-level causal-control and information-flow metrics for the 4 functional modules; the PA module shows the highest micro-to-macro flow efficiency and macro-level control metric. (B) Principal-component (PC) representation of the 14-dimensional exploratory micro-state and 4-dimensional macro-state representations. (C) Paired bootstrap comparisons of model-derived metrics between micro-symptom and macro-module descriptions; asterisks (** and ***) denote the displayed bootstrap significance convention. (D) Dependence of the model-derived effect size on description scale, with the largest value at the 4-module scale. The 14 micro nodes comprise 12 core indicators plus information seeking and fatigue. AP: abdominal pain; AV: avoidance; BL: bloating; BU: bowel urgency; CA: cognitive anxiety; CT: catastrophizing; ER: emotion regulation; FA: fatigue; GS: gut-specific symptoms; HW: health worry; IS: information seeking; NA: negative affect; PF: psychosocial factors; RU: rumination; SA: somatic anxiety; SC: social conflict; WS: work stress.

### TVEM

The TVEM used 6660 daily observations from 74 patients. The archived fast restricted maximum likelihood evaluation output for the canonical TVEM input reported 64.4% deviance explained (adjusted *R*^²^=0.642) for the full module-control model and 45.6% (adjusted *R*^²^=0.453) for the full causal-emergence model. The subgroup fits were reported 64.7% and 45.7%, respectively; the basic module-control fit was 62.9%. TVEM fit metrics, residual diagnostics, dependence parameters, and basis/scaled t-sensitivity results are reported in Tables S2 and S8 in Multimedia Appendix 1. Figure 6 reproduces the original 3-part presentation: panel A displays the adjusted population trajectories for module-control strength and the causal-emergence summary; panel B displays subtype-specific module-control trajectories; and panel C displays the adjusted covariate-effect curves for disease activity and HADS. Lines are model-derived estimates with pointwise 95% intervals. The treatment-initiation marker is descriptive and is not an intervention contrast. The model fit and complexity metrics for the basic, full, and subgroup TVEM specifications are summarized in Table 2.

**Figure 6.**
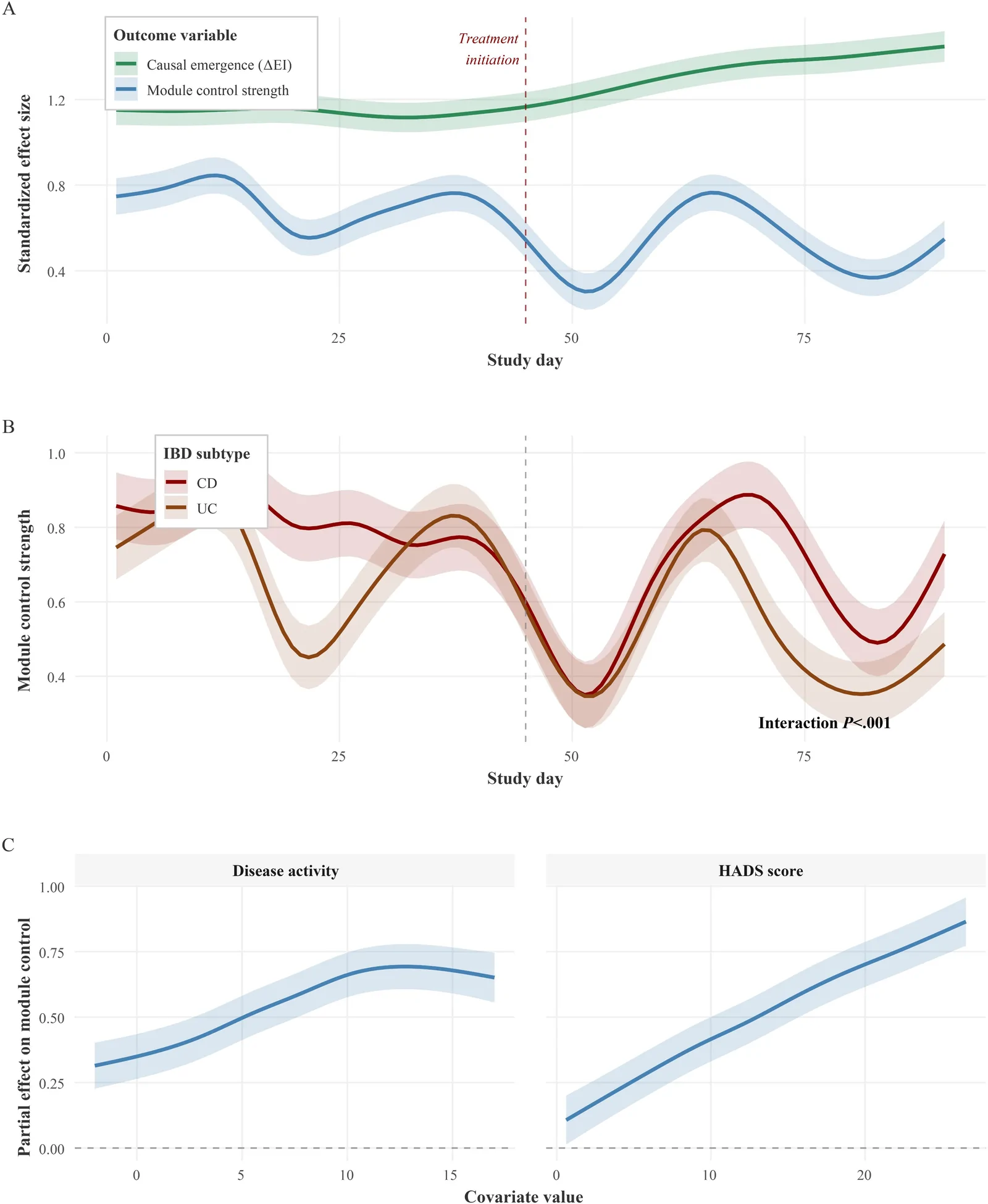
Exploratory time-varying effect model results. (A) Adjusted population trajectories for module-control strength and the causal-emergence summary. (B) Subtype-specific module-control trajectories for Crohn disease (CD) and ulcerative colitis (UC). (C) Adjusted covariate-effect curves for disease activity and Hospital Anxiety and Depression Scale (HADS). Shaded bands are pointwise 95% intervals. EI: effective information.

**Table 2.** Exploratory TVEM^a^ model fit and complexity metrics^b^.

Model	AIC^c^	BIC^d^	Deviance explained (%)	Adjusted *R*²	Total EDF^e^	Significant smooths
Model 1: Basic (module control)	–10331.3	–9757.7	62.9	0.625	82.2	2
Model 1: Full (module control)	–10682.6	–10397.4	64.4	0.642	21.5	4
Model 2: Full (causal emergence)	3054.5	3371.2	45.6	0.453	23.2	2
Model 1: Subgroup (module control)	–10714.3	–10343.3	64.7	0.644	34.2	6
Model 2: Subgroup (causal emergence)	3057.3	3432.9	45.7	0.453	31.4	3

a TVEM: time-varying effect model.

b The basic model uses the lower-dimension (*k*=5) module-control sensitivity fit; the full models use the canonical *k*=7 specification; subgroup models include subtype-specific smooth functions.

c AIC: Akaike information criterion.

d BIC: information criterion.

e EDF: effective degrees of freedom.

## Discussion

### Principal Findings

In this 90-day prospective cohort of 74 patients with IBD, intensive daily monitoring identified a reproducible temporal organization of stress and symptoms that is not visible in intermittent clinic assessments. The prespecified cycle-end contrast was the clearest empirical signal: mean stress was 0.629 points higher during study days 26 to 30 than during the remaining days (95% CI 0.550‐0.708; *P*<.001), and 71 of 74 (95.9%) participants showed a positive participant-specific contrast. This finding is important because it establishes a temporal regularity without requiring the stronger claim that IBD has a universal monthly rhythm. Against this temporal background, the exploratory network analyses converged on a structured sequence linking PF, ER, PA, and GS. PF-to-ER and PA-to-GS interfaces were the densest cross-module connections, while symptom-to-PA feedback and increasing within-module density suggested that the observed system was both directional and reciprocal. Finally, the multiscale analysis showed its largest model-derived effect at the 4-module description and highest micro-to-macro flow efficiency and macro-level control for PA. The secondary time-varying models were consistent with changing organization over the follow-up period, but their fit statistics remain descriptive. Taken together, the study provides a compact, clinically interpretable map of temporal coordination.

### Temporal Organization of Stress and Symptoms

The cycle-end result gives the framework a concrete behavioral anchor; it was based on a prespecified window and a participant-level mixed model, and the direction was concordant across nearly the entire cohort. The most defensible interpretation is therefore that perceived stress was temporally patterned in this sample, with a recurring increase near the end of each 30-day observation cycle. Such organization could arise from work schedules, social obligations, health-related planning, or other recurring contexts. The distinction matters clinically. A patient whose stress and abdominal symptoms repeatedly co-occur around a predictable period may benefit from anticipatory assessment and support, whereas a patient with no such pattern should not be assigned to the same schedule. Digital phenotyping makes this distinction empirically approachable by resolving within-person timing rather than treating stress as a single baseline covariate [28-31].

The individual and cohort summaries also show why temporal heterogeneity should be retained rather than averaged away. Symptom burden had a high mean and very low dispersion in the aggregate, whereas stressors and psychophysiological indicators varied more across patients. A high average symptom score can therefore coexist with different routes through which stress is experienced, regulated, and reported. The lagged network should be interpreted in that context: the PF-to-ER organization is compatible with stress being expressed through rumination, catastrophizing, avoidance, or information seeking before appearing alongside gut symptoms, but it does not prove that any one node transmits a biological signal. Likewise, the absence of a single population-level coefficient for every stress-cognition pair would not invalidate the cycle-end contrast; a common timing pattern can coexist with heterogeneous pairwise associations. This is precisely the value of reporting patient support, signed direction agreement, and cluster-bootstrap uncertainty rather than presenting one pooled edge as a universal mechanism.

### Multiscale Architecture of Stress-Symptom Coupling

The 4-module representation organizes the results around interfaces that are recognizable to clinicians: PF contains work stress, health worry, and social conflict; ER contains rumination, catastrophizing, avoidance, and information seeking; PA contains cognitive anxiety, somatic anxiety, negative affect, and fatigue; and GS contains abdominal pain, bloating, and bowel urgency. The highest PF-to-ER density indicates that psychosocial exposures were most consistently connected to ER variables in the fitted network. The next most prominent interface, PA-to-GS, places affective and somatic amplification close to the symptoms that patients bring to care. Feedback from GS to PA is equally informative: abdominal sensations may become inputs to the same affective-somatic configuration that accompanies symptom escalation. This topology is more consistent with reciprocal brain-gut coupling than with a one-way stress-to-symptom chain, while remaining agnostic about the underlying physiology [29-32].

The increase in within-module density during follow-up and the relatively high modularity of ER and PA add a second layer of interpretation. Variables within these modules increasingly covaried as a functional unit, yet the modules remained distinguishable from one another. In network terms, the data suggest coordinated clusters and bottlenecks rather than 12 independent symptom scores; in clinical terms, a change in one affective or fatigue-related measure may be accompanied by changes in several neighboring measures. The PA label is deliberately descriptive. It denotes the measured grouping of cognitive anxiety, somatic anxiety, negative affect, and fatigue and should not be equated with a validated neurobiological central-sensitization construct. Existing work on the brain-gut axis and central sensitization provides a biologically plausible context for this grouping [31,32], but this study did not measure nociceptive processing, neural activity, or peripheral mediators. Its contribution is to identify a candidate integrative representation that can be tested against those measures.

### Why the Module Scale Matters

The effective-information analysis helps explain why the module-level description was useful: when 14 daily series were compressed into 4 prespecified modules, the model-derived representation showed greater micro-to-macro flow efficiency and macro-level control for PA, and the largest effect size occurred at the 4-module scale. It indicates that, within the chosen state encoding and transition model, correlated indicators could be summarized with less apparent noise and more coherent transition information at the module scale. In other words, the module may function as an information bottleneck: many small fluctuations are represented by a smaller number of clinically legible states. This is closely aligned with the motivation of network medicine, in which patterns of interaction can be more informative than isolated variables [27,33,34]. It also provides a principled reason to report both micro-level and module-level results rather than selecting the most striking metric post hoc.

The multiscale result should therefore be understood as a hypothesis about representation. If the PA module is a robust integrative unit, its summary state should replicate in independent cohorts, remain stable under alternative scaling and missing-data procedures, and correspond to external measures of symptom amplification or brain-gut function. Conversely, if the apparent gain disappears when the state definition, lag window, or cohort changes, the finding will have identified a property of this analytic representation rather than a general feature of IBD. This distinction turns the causal-emergence terminology into a testable methodological proposition and avoids treating a model-derived quantity as a treatment target.

### Temporal Reorganization and Disease-State Context

The time-varying analyses extend the cross-sectional network picture by showing that the organization of these relationships was not constant over 90 days. Within the canonical input, the full module-control model explained 64.4% of deviance (adjusted *R*^2^=0.642), and the corresponding causal-emergence model explained 45.6% (adjusted *R*^2^=0.453); subgroup and lower-dimensional fits were similar. These values describe how well the specified smooth functions represented the supplied daily model-derived outcomes. They do not show that the network became more effective due to treatment, nor do they show that a higher curve predicts recovery. The descriptive treatment-initiation marker in Figure 6 should be read accordingly.

The upward trajectory in within-module coordination and the moderation by baseline disease activity are nevertheless clinically informative observations. They suggest that the same measured stress-symptom architecture may be expressed at different tempos in patients entering follow-up with different inflammatory burdens. Several processes could produce this pattern, including inflammation-driven peripheral input, treatment exposure, adaptation to monitoring, changes in symptom appraisal, or regression toward the mean. The important result is not that one of these explanations has been proven, but that disease state is a plausible context variable that should be carried into future longitudinal designs. This complements treat-to-target principles, which distinguish inflammatory control from patient-reported symptom control and require both to be monitored [35,36].

### Clinical Meaning as a Testable Hypothesis

The immediate clinical value of this work is, therefore, phenotyping rather than prescribing. A gastroenterologist could use repeated measures to ask whether a patient’s stress, affective, fatigue, and gut-symptom signals form a stable temporal pattern, whether the pattern changes with inflammatory activity, and which time windows merit closer review. Such information could complement, rather than replace, objective disease assessment and routine symptom measures. It may also help explain why patients with apparently controlled inflammation continue to report substantial symptoms, a recognized challenge in IBD care [35,36].

The framework also creates a disciplined path toward intervention research. Cognitive behavioral and mindfulness-based approaches are reasonable candidates because prior studies have examined psychological and functional GI outcomes [37,38], and individualized time-series models provide tools for studying person-specific change [39,40]. In this study, however, no psychological, behavioral, or pharmacologic intervention was assigned, and no analysis tested whether changing PF, ER, PA, or GS improved symptoms, quality of life, inflammation, or treatment response. Consequently, the network and module metrics should be used to generate prespecified hypotheses for pragmatic or micro-randomized trials, not to justify a module-specific therapy. Future studies can test whether an intervention changes the predicted edge, module state, or clinically meaningful end point, with inflammatory and patient-reported outcomes assessed together. That progression preserves the potential clinical relevance of digital phenotyping while keeping exploratory association, mechanistic explanation, and therapeutic efficacy analytically distinct.

### Limitations

This study has 4 principal boundaries. The 74 participants were recruited from a single region, so the patient-level support and module summaries require replication in larger, multicenter cohorts. Although the repeated daily records improve temporal resolution, they did not increase the independent sample size beyond 74 participants. Most inputs were self-reported, and the observational design leaves room for unmeasured influences such as treatment changes, sleep, diet, adherence, infection, and inflammatory biomarkers. The prespecified cycle-end contrast, PCMCI links, module-control metrics, effective-information summaries, and TVEM trajectories were analyzed as exploratory or descriptive results, with code and preprocessing details supplied for reproduction. Longer follow-up, multimodal measurement, external validation, and intervention-based causal tests are needed before any network feature is used for clinical decision-making.

### Conclusions

Ninety days of daily monitoring among 74 patients with IBD revealed a reproducible cycle-end stress contrast and a model-derived multiscale organization linking PF, ER, PA indicators, and GS. The PA module was the most informative integrative representation in the selected effective-information analysis, while temporal and disease-state patterns indicated that this organization can change during follow-up. These findings support digital phenotyping as a way to formulate clinically grounded, person-specific hypotheses about stress-symptom coordination.

## Supplementary material

10.2196/95854 Multimedia Appendix 1 Figure and tables describing participant-day stress distributions, the primary cycle-end stress contrast, variable definitions and scoring rules, data availability and missingness, preprocessing and model specifications, and sensitivity and diagnostic analyses.

## Abbreviations

AR(1): first-order autoregressive
CERQ: Cognitive Emotion Regulation Questionnaire
ER: emotion regulation
GI: gastrointestinal
GS: gut-specific symptom
HADS: Hospital Anxiety and Depression Scale
HISBS: Health Information Seeking Behavior Scale
IBD: inflammatory bowel disease
IBD-SI: Inflammatory Bowel Disease Symptom Score Scale
MFI-20: 20-item Multidimensional Fatigue Inventory
mHealth: mobile health
PA: psychophysiological amplification
PANAS: Positive and Negative Affect Schedule
ParCorr: partial correlation
PCMCI: Peter and Clark Momentary Conditional Independence
PF: psychosocial factor
PSS-10: 10-item Perceived Stress Scale
SES-CD: Simplified Endoscopic Activity Score for Crohn Disease
TVEM: time-varying effect model
