# Supplementary material for: Stress-Symptom Interaction in Inflammatory Bowel Disease: Longitudinal Digital Phenotyping Study
Source: JMIR Med Inform. 2026 Oct 22;14:e95854. doi: 10.2196/95854 (PMC13717339; doi:10.2196/95854)
Supplement: Supplementary file 1 [file medinform-v14-e95854-s001.docx]

Multimedia Appendix 1

**Figure** S1. Participant-day stress distributions across the 90-day study period and prespecified cycle-end windows. Each translucent point represents a participant-day observation and is colored by stress score (blue-to-red scale). Larger white-outlined points trace the daily group mean, with fill encoding the daily SD (yellow-to-purple scale). Open inverted triangles mark the end of each 30-day study cycle, dashed annotations identify the prespecified cycle-end window (cycle days 26-30), and the scale bar represents 15 days along the temporal trajectory.

**Table** S1. Primary month-end stress contrast and ancillary stress-cognition summaries

| **Analysis** | **Descriptive result** | **Effect estimate or distribution** | **P value** |
| --- | --- | --- | --- |
| Sample and window | 74 participants; 6,660 person-days; month-end defined as cycle days 26-30 | — | — |
| Stress score, mean | Month-end 4.789; non-month-end 4.160 | Within-person Δ=0.629 (95% CI 0.550 to 0.708) | <.001 |
| Mixed-effects model | Stress score modeled as a function of month-end window and study day, with participant random intercept | β_month-end=0.608 (95% CI 0.553 to 0.663) | <.001 |
| Individual month-end − non-month-end differences | 71/74 positive (95.9%); 3/74 negative (4.1%) | Min=−0.146; Q1=0.430; median=0.615; Q3=0.881; max=1.369 | — |
| Individual stress-cognition slopes | 37/74 positive (50.0%); 37/74 negative (50.0%) | Mean=0.035; median=0.044; IQR=−0.499 to 0.576 | Not applicable |
| Group-level Stress-Cognition Association | Model-derived network metric=0.000 | — | Not applicable |
| Pooled stress-cognition model coefficient | — | β_stress=−0.057 (95% CI −0.192 to 0.079) | .413 |

**Table** S2. Variable definitions, sources, measurement timing, scoring ranges, and analysis roles

| **Variable / definition** | **Source / timing** | **Observed or source range** | **Transformation / scaling** | **Reverse coding** | **Composite formula** | **Missing rule** | **Analysis role** |
| --- | --- | --- | --- | --- | --- | --- | --- |
| Work Stress; item IDs WS01-WS10; 10 daily item(s) | Perceived Stress Scale (PSS-10); daily; item IDs WS01-WS10 | 0-4 | Raw app score for cycle contrast; patient-level z for network | 4 (4 - raw); 5 (4 - raw); 7 (4 - raw); 8 (4 - raw) | Prorated sum = (sum of valid item scores × 10) / number of valid items; then apply prorated_sum/4. | Composite missing when fewer than 6 of 10 items are valid; otherwise computed from valid items; no carry-forward. In PCMCI only, variable-level missingness <=5% permits internal linear interpolation for gaps <=2 days. | Core PF; denominator and exploratory 14-series input |
| Health Worry; item IDs HW01-HW03; 3 daily item(s) | Health Worry Scale; daily; item IDs HW01-HW03 | 0-4 | Within-patient z for network | None | Mean of valid item scores; then apply item_mean*2.5. | Composite missing when fewer than 2 of 3 items are valid; otherwise computed from valid items; no carry-forward. In PCMCI only, variable-level missingness <=5% permits internal linear interpolation for gaps <=2 days. | Core PF; denominator and network input |
| Social Conflict; item IDs SC01-SC03; 3 daily item(s) | Social Conflict Scale; daily; item IDs SC01-SC03 | 0-4 | Within-patient z for network | None | Mean of valid item scores; then apply item_mean*2.5. | Composite missing when fewer than 2 of 3 items are valid; otherwise computed from valid items; no carry-forward. In PCMCI only, variable-level missingness <=5% permits internal linear interpolation for gaps <=2 days. | Core PF; denominator and network input |
| Rumination; item IDs RUM01-RUM04; 4 daily item(s) | Rumination Scale; daily; item IDs RUM01-RUM04 | 1-5 | Patient-level z for network | None | (prorated_sum-4)/1.6 | Composite missing when fewer than 3 of 4 items are valid; otherwise computed from valid items; no carry-forward. In PCMCI only, variable-level missingness <=5% permits internal linear interpolation for gaps <=2 days. | Core ER; denominator and network input |
| Catastrophizing; item IDs CAT01-CAT04; 4 daily item(s) | Catastrophizing Scale; daily; item IDs CAT01-CAT04 | 1-5 | Patient-level z for network | None | (prorated_sum-4)/1.6 | Composite missing when fewer than 3 of 4 items are valid; otherwise computed from valid items; no carry-forward. In PCMCI only, variable-level missingness <=5% permits internal linear interpolation for gaps <=2 days. | Core ER; denominator and network input |
| Avoidance; item IDs AVD01-AVD04; 4 daily item(s) | Avoidance Scale; daily; item IDs AVD01-AVD04 | 1-5 | Within-patient z for network | None | (item_mean-1)*2.5 | Composite missing when fewer than 3 of 4 items are valid; otherwise computed from valid items; no carry-forward. In PCMCI only, variable-level missingness <=5% permits internal linear interpolation for gaps <=2 days. | Core ER; denominator and network input |
| Information Seeking; item IDs INF01-INF04; 4 daily item(s) | Information Seeking Scale; daily; item IDs INF01-INF04 | 1-5 | Patient-level z for exploratory network only | None | (item_mean-1)*2.5 | Composite missing when fewer than 3 of 4 items are valid; otherwise computed from valid items; no carry-forward. In PCMCI only, variable-level missingness <=5% permits internal linear interpolation for gaps <=2 days. | Auxiliary ER input only for exploratory PCMCI/module/causal-emergence |
| Cognitive Anxiety; item IDs CGA01-CGA04; 4 daily item(s) | Cognitive Anxiety Scale; daily; item IDs CGA01-CGA04 | 0-3 | Patient-level z for network | 4 (3 - raw) | Prorated sum = (sum of valid item scores × 4) / number of valid items; then apply prorated_sum/1.2. | Composite missing when fewer than 3 of 4 items are valid; otherwise computed from valid items; no carry-forward. In PCMCI only, variable-level missingness <=5% permits internal linear interpolation for gaps <=2 days. | Core PA; denominator and network input |
| Somatic Anxiety; item IDs SMA01-SMA03; 3 daily item(s) | Somatic Anxiety Scale; daily; item IDs SMA01-SMA03 | 0-3 | Within-patient z for network | None | Prorated sum = (sum of valid item scores × 3) / number of valid items; then apply prorated_sum/0.9. | Composite missing when fewer than 2 of 3 items are valid; otherwise computed from valid items; no carry-forward. In PCMCI only, variable-level missingness <=5% permits internal linear interpolation for gaps <=2 days. | Core PA; denominator and network input |
| Negative Affect; item IDs NA01-NA10; 10 daily item(s) | Negative Affect Scale; daily; item IDs NA01-NA10 | 1-5 | Patient-level z for network | None | (prorated_sum-10)/4 | Composite missing when fewer than 8 of 10 items are valid; otherwise computed from valid items; no carry-forward. In PCMCI only, variable-level missingness <=5% permits internal linear interpolation for gaps <=2 days. | Core PA; denominator and network input |
| Fatigue; item IDs FAT01-FAT20; 20 daily item(s) | Fatigue Scale; daily; item IDs FAT01-FAT20 | 1-5 | Patient-level z for exploratory network only | 1 (6 - raw); 3 (6 - raw); 4 (6 - raw); 6 (6 - raw); 7 (6 - raw); 11 (6 - raw); 12 (6 - raw); 15 (6 - raw); 20 (6 - raw) | (prorated_sum-20)/8 | Composite missing when fewer than 16 of 20 items are valid; otherwise computed from valid items; no carry-forward. In PCMCI only, variable-level missingness <=5% permits internal linear interpolation for gaps <=2 days. | Auxiliary PA input only for exploratory PCMCI/module/causal-emergence |
| Abdominal Pain; item ID PAIN01; 1 daily item(s) | Gastrointestinal Symptom Scale; daily; item ID PAIN01 | 0-10 | Within-patient z for network | None | Single valid item value; no averaging or weighting. | Composite missing when fewer than 1 of 1 items are valid; otherwise computed from valid items; no carry-forward. In PCMCI only, variable-level missingness <=5% permits internal linear interpolation for gaps <=2 days. | Core GS; denominator and network input |
| Bloating; item ID BLOAT01; 1 daily item(s) | Gastrointestinal Symptom Scale; daily; item ID BLOAT01 | 0-10 | Within-patient z for network | None | Single valid item value; no averaging or weighting. | Composite missing when fewer than 1 of 1 items are valid; otherwise computed from valid items; no carry-forward. In PCMCI only, variable-level missingness <=5% permits internal linear interpolation for gaps <=2 days. | Core GS; denominator and network input |
| Bowel Urgency; item ID URG01; 1 daily item(s) | Gastrointestinal Symptom Scale; daily; item ID URG01 | 0-10 | Within-patient z for network | None | Single valid item value; no averaging or weighting. | Composite missing when fewer than 1 of 1 items are valid; otherwise computed from valid items; no carry-forward. In PCMCI only, variable-level missingness <=5% permits internal linear interpolation for gaps <=2 days. | Core GS; denominator and network input |
| Study day | Study schedule index; daily | 0-89 | No scaling; sorted within patient | Not applicable | Not applicable | Required; no endpoint filling | Time index for lagging, cycles, transitions and TVEM |
| Module-control strength | Derived from retained PCMCI links | Model-derived scale | No raw-score scaling | Not applicable | Thresholded graph summary | Complete-case derived output | Exploratory TVEM outcome |
| Causal emergence / adjusted EI | Derived from 14-series transitions | Bits / model-derived scale | Within-patient rank bins; smoothing 0.50 | Not applicable | Macro EI minus micro EI | Complete-case derived output | Exploratory TVEM outcome |

Note: Item IDs, response ranges, reverse-scoring rules, minimum valid item counts, and composite formulas are taken from the CRF scoring specification. All daily composites are reported on a 0-10 scale.

**Table** S3. Record availability and missingness in the supplied analysis files

| **Module** | **Variable** | **Available** | **Missing, n (%)** | **Completion** | Missing days/patient, median (IQR; all 74 patients) | **Observed range** | **Patients >5% missing** |
| --- | --- | --- | --- | --- | --- | --- | --- |
| PF | Work_Stress | 6660 | 0 (0.0%) | 100.0% | 0.0 (0.0-0.0) | 0.000-10.000 | 0 |
| PF | Health_Worry | 6644 | 16 (0.2%) | 99.8% | 0.0 (0.0-0.0) | 0.000-10.000 | 0 |
| PF | Social_Conflict | 6653 | 7 (0.1%) | 99.9% | 0.0 (0.0-0.0) | 0.000-10.000 | 0 |
| ER | Rumination | 6639 | 21 (0.3%) | 99.7% | 0.0 (0.0-0.0) | 0.000-10.000 | 0 |
| ER | Catastrophizing | 6636 | 24 (0.4%) | 99.6% | 0.0 (0.0-0.0) | 0.000-10.000 | 0 |
| ER | Avoidance | 6628 | 32 (0.5%) | 99.5% | 0.0 (0.0-1.0) | 0.000-10.000 | 0 |
| PA | Cog_Anxiety | 6636 | 24 (0.4%) | 99.6% | 0.0 (0.0-1.0) | 0.000-10.000 | 0 |
| PA | Soma_Anxiety | 6649 | 11 (0.2%) | 99.8% | 0.0 (0.0-0.0) | 0.000-10.000 | 0 |
| PA | Negative_Affect | 6650 | 10 (0.2%) | 99.8% | 0.0 (0.0-0.0) | 0.000-10.000 | 0 |
| GS | Abdominal_Pain | 6359 | 301 (4.5%) | 95.5% | 4.0 (3.0-5.0) | 0.000-10.000 | 28 |
| GS | Bloating | 6338 | 322 (4.8%) | 95.2% | 4.0 (3.0-6.0) | 0.000-10.000 | 31 |
| GS | Bowel_Urgency | 6336 | 324 (4.9%) | 95.1% | 4.0 (3.0-6.0) | 0.000-10.000 | 32 |
| ER | Info_Seeking | 6635 | 25 (0.4%) | 99.6% | 0.0 (0.0-1.0) | 0.000-10.000 | 0 |
| PA | Fatigue | 6660 | 0 (0.0%) | 100.0% | 0.0 (0.0-0.0) | 0.000-10.000 | 0 |
| Total | 12 core variables | 78828 | 1092 (1.4%) | 98.6% | Not pooled across variables | Not pooled | Not pooled |
| Auxiliary | Information Seeking + Fatigue | 13295 | 25 (0.2%) | 99.8% | Reported per variable above | Not pooled | Not pooled |

**Table** S4. Preprocessing, scaling, and primary model parameters

| **Stage** | **Rule / parameter** | **Exact implementation** | **Purpose / affected analysis** |
| --- | --- | --- | --- |
| Data integrity | Range and logic checks | App-enforced score limits; verify unique participant-day keys; sort by participant and study day. | All analyses |
| Raw denominator | Planned variable-days | 74 participants x 90 days x 12 core variables = 79,920. | Reporting denominator |
| Interpolation | Raw variable missingness <=5% | Linear interpolation only for internal gaps of at most 2 observations within patient; no cross-patient or end-point carry-forward. | Longitudinal network inputs |
| Residual gaps | After policy | Remaining missing values are removed by complete-case analysis within the relevant stage; no time point is silently carried forward. | All longitudinal analyses |
| Variable eligibility | Completion >=92% | Variables below the threshold are flagged in the audit; auxiliary variables are reported separately from the 12-core denominator. | Core and auxiliary input audit |
| Scaling | Within-person standardization | After missing-data handling, z=(x-person mean)/(person SD); exclude or flag zero-variance series. Retain raw scores for descriptive cycle-end contrasts. | PCMCI, module-control inputs, selected TVEM covariates |
| Smoothing | No smoothing in inferential analyses | Any 7-day moving average is visualization-only and must not enter PCMCI, module-control, causal-emergence, or TVEM fitting. | Descriptive figures only |
| PCMCI | ParCorr; tau=0,1,2; alpha=.05 | Analytic partial-correlation test; patient-level BH q values; patient boundaries preserved; no AR(2) correction. | Exploratory conditional-association networks |
| Module control | Four prespecified modules | PF, ER, PA, and GS; network metrics derived from retained PCMCI links and integrated-control nodes. | Module-control analysis |
| Causal emergence | Two rank categories per micro variable | Rounded module-mean macro states; additive smoothing 0.50; patient bootstrap, within-patient permutation, and 2/3-category sensitivity. | Exploratory effective-information contrast |
| TVEM | Primary fixed day knots 0, 10, 25, 50, 65, 75, 89 (cycle landmarks 25, 50, 75) | mgcv::bam; patient random intercept; patient-level AR(1) working correlation with rho recorded; lower/higher basis and scaled-t sensitivity. | Secondary exploratory model |
| Inference | Patient is the independent unit | Resampling, interpolation, lagging, transitions, and uncertainty calculations preserve participant clusters. | All inferential and sensitivity analyses |

**Table** S5. PCMCI lag/alpha sensitivity and patient-level stability results

| **tau max** | **alpha** | **Eligible patients** | **Tested links** | **Significant links** | **Consensus edges** | **Edges support >=.30** | **Median support** |
| --- | --- | --- | --- | --- | --- | --- | --- |
| 1 | 0.01 | 64 | 25088 | 1026 | 185 | 20 | 0.0312 |
| 1 | 0.05 | 64 | 25088 | 1438 | 265 | 28 | 0.0312 |
| 1 | 0.10 | 64 | 25088 | 1793 | 298 | 30 | 0.0312 |
| 2 | 0.01 | 64 | 37632 | 667 | 152 | 6 | 0.0156 |
| 2 | 0.05 | 64 | 37632 | 952 | 240 | 12 | 0.0156 |
| 2 | 0.10 | 64 | 37632 | 1162 | 290 | 22 | 0.0156 |
| 3 | 0.01 | 64 | 50176 | 510 | 128 | 2 | 0.0156 |
| 3 | 0.05 | 64 | 50176 | 614 | 175 | 6 | 0.0156 |
| 3 | 0.10 | 64 | 50176 | 761 | 251 | 6 | 0.0156 |

**Table** S6. Module-control threshold sensitivity results

| **Support threshold** | **Absolute effect threshold** | **Edge count** | **Density** | **Driver count** |
| --- | --- | --- | --- | --- |
| 0.20 | 0.05 | 30 | 0.165 | 0 |
| 0.20 | 0.10 | 30 | 0.165 | 0 |
| 0.20 | 0.20 | 30 | 0.165 | 0 |
| 0.30 | 0.05 | 18 | 0.099 | 4 |
| 0.30 | 0.10 | 18 | 0.099 | 4 |
| 0.30 | 0.20 | 18 | 0.099 | 4 |
| 0.40 | 0.05 | 6 | 0.033 | 10 |
| 0.40 | 0.10 | 6 | 0.033 | 10 |
| 0.40 | 0.20 | 6 | 0.033 | 10 |
| 0.50 | 0.05 | 0 | 0.000 | 14 |
| 0.50 | 0.10 | 0 | 0.000 | 14 |
| 0.50 | 0.20 | 0 | 0.000 | 14 |

**Table** S7. Causal-emergence bootstrap, permutation, and state-resolution results

| **Component / resolution** | **Estimate** | **95% interval / null SD** | **Repetitions / transitions** | **Interpretation** |
| --- | --- | --- | --- | --- |
| Patient bootstrap: micro_ei_bits | 0.0042 | 0.0033 to 0.0056 | 200 patient-cluster resamples | Bootstrap does not refit upstream PCMCI |
| Patient bootstrap: macro_ei_bits | 0.2982 | 0.2654 to 0.3449 | 200 patient-cluster resamples | Bootstrap does not refit upstream PCMCI |
| Patient bootstrap: delta_ei_bits | 0.2940 | 0.2613 to 0.3399 | 200 patient-cluster resamples | Bootstrap does not refit upstream PCMCI |
| Temporal permutation: delta EI | 0.2355 | null mean 0.0631; SD 0.0063; upper-tail P=0.0020 | 500 within-patient permutations | Preserves patient marginal states |
| State resolution: 2 categories | 0.2355 | Not applicable | 2909 transitions across 74 patients | Sensitivity result |
| State resolution: 3 categories | 0.2946 | Not applicable | 2909 transitions across 74 patients | Sensitivity result |

**Table** S8. TVEM fit, dependence, diagnostic, and sensitivity results

| **Model / component** | **Basis / distribution** | **rho or sensitivity** | **AIC** | **BIC** | **Deviance explained / interpretation** |
| --- | --- | --- | --- | --- | --- |
| module | mgcv::bam; cubic spline; patient random intercept | archived canonical fit; fixed knots | -10682.6 | -10397.4 | 64.4%; canonical full module-control fit |
| emergence | mgcv::bam; cubic spline; patient random intercept | archived canonical fit; fixed knots | 3054.5 | 3371.2 | 45.6%; canonical full causal-emergence fit |
| module_subtype | Subtype-specific cubic spline fit | subtype-specific smooths | -10714.3 | -10343.3 | 64.7%; subgroup fit |
| emergence_subtype | Subtype-specific cubic spline fit | subtype-specific smooths | 3057.3 | 3432.9 | 45.7%; subgroup fit |
| module_basic | Lower-dimension cubic spline fit | basic module-control sensitivity | -10331.3 | -9757.7 | 62.9%; sensitivity fit |
| emergence_k5 | Sensitivity refit | Basis or scaled-t alternative | -8631.9 | -8268.7 | - |
| module_k9 | Sensitivity refit | Basis or scaled-t alternative | -16226.4 | -16120.9 | - |
| emergence_k9 | Sensitivity refit | Basis or scaled-t alternative | -8635.2 | -8255.2 | - |
| module_scaled_t | Sensitivity refit | Basis or scaled-t alternative | -17959.6 | -17850.8 | - |
| emergence_scaled_t | Sensitivity refit | Basis or scaled-t alternative | -8728.0 | -8333.7 | - |

**Table** S9. Patient-level robustness of the cycle-end stress contrast

| **Analysis** | **Patient-level unit / repetitions** | **Estimate** | **95% CI or range** | **P value / interpretation** |
| --- | --- | --- | --- | --- |
| Primary within-person contrast | 74 patient-specific end-window minus non-window means | 0.629 | 0.550 to 0.708 | Paired t P=2.29e-25; 71/74 positive |
| Patient-cluster bootstrap | 500 resamples of 74 patients | Mean 0.634; median 0.633 | 0.560 to 0.717 | Empirical two-sided P<.002; patients resampled, no model refit |
| Leave-one-patient-out | 74 omissions | Mean 0.629 | 0.619 to 0.640 | All omissions retained a positive contrast |
| Window sensitivity | 3-, 5-, and 7-day windows | 0.664; 0.629; 0.472 | All positive | Window definition changes magnitude, not direction |
| Independent-sample interpretation | Patient, not person-day, is inferential unit | 74 patients; 6,660 daily records | Effective independent N=74 | Repeated measures improve temporal resolution but do not increase independent sample |

Note. The end-window was defined as fixed study-cycle days 26-30; the primary outcome was raw stress and the comparison was all non-window days.
